# A Multi-Period Optimization Model for Service Providers Using Online Reservation Systems: An Application to Hotels

**DOI:** 10.1371/journal.pone.0128574

**Published:** 2015-07-06

**Authors:** Ming Xu, Yan Jiao, Xiaoming Li, Qingfeng Cao, Xiaoyang Wang

**Affiliations:** 1 College of Tourism and Service Management, Nankai University, Tianjin, 300074, China; 2 Institute of Urban and Regional Economics, Nankai University, Tianjin, 300071, China; 3 School of Mathematics and Statistics, Beijing Institute of Technology, Beijing 100081, China; Nankai University, CHINA

## Abstract

This paper presents a multi-period optimization model for high margin and zero salvage products in online distribution channels with classifying customers based on number of products required. Taking hotel customers as an example, one is regular customers who reserve rooms for one day, and the other is long term stay (LTS) customers who reserve rooms for a number of days. LTS may guarantee a specific amount of demand and generate opportunity income for a certain number of periods, meanwhile with risk of punishment incurred by overselling. By developing an operational optimization model and exploring the effects of parameters on optimal decisions, we suggest that service providers should make decisions based on the types of customers, number of products required, and duration of multi-period to reduce the loss of reputation and obtain more profit; at the same time, multi-period buying customers should buy products early. Finally, the paper conducts a numerical experiment, and the results are consistent with prevailing situations.

## Introduction

The rapid development of E-commerce and Mobile-business has increased the number of consumers who are willing to purchase products and services online (e.g., flight tickets, hotel rooms, and car rentals). This service allows consumers the convenience of making reservations anytime and anywhere. In a survey of 249 leisure travelers in the hotel industry, more than half made their bookings online [1]. Subsequently, service providers have used the online reservation systems (ORS) to facilitate the reservations of consumers who have Internet access.

In practice, the number of products required by each customer is different. Specifically, regular customers generally reserve one product, such as one flight ticket or one hotel room for one day; other customers may need more products, such as round-trip ticket and hotel rooms for long-term stay (LTS). Taking hotels as an example, LTS in a hotel is common in reality as the economy develops and tourism increases in popularity [2–7]. For instance, people will stay in a hotel in a holiday resort (e.g., Hawaii) for several days to enjoy their vacation. The request for LTS from employees who are assigned by a company to establish a branch in a city or conduct business for several days is another common example.

**Fig 1:**
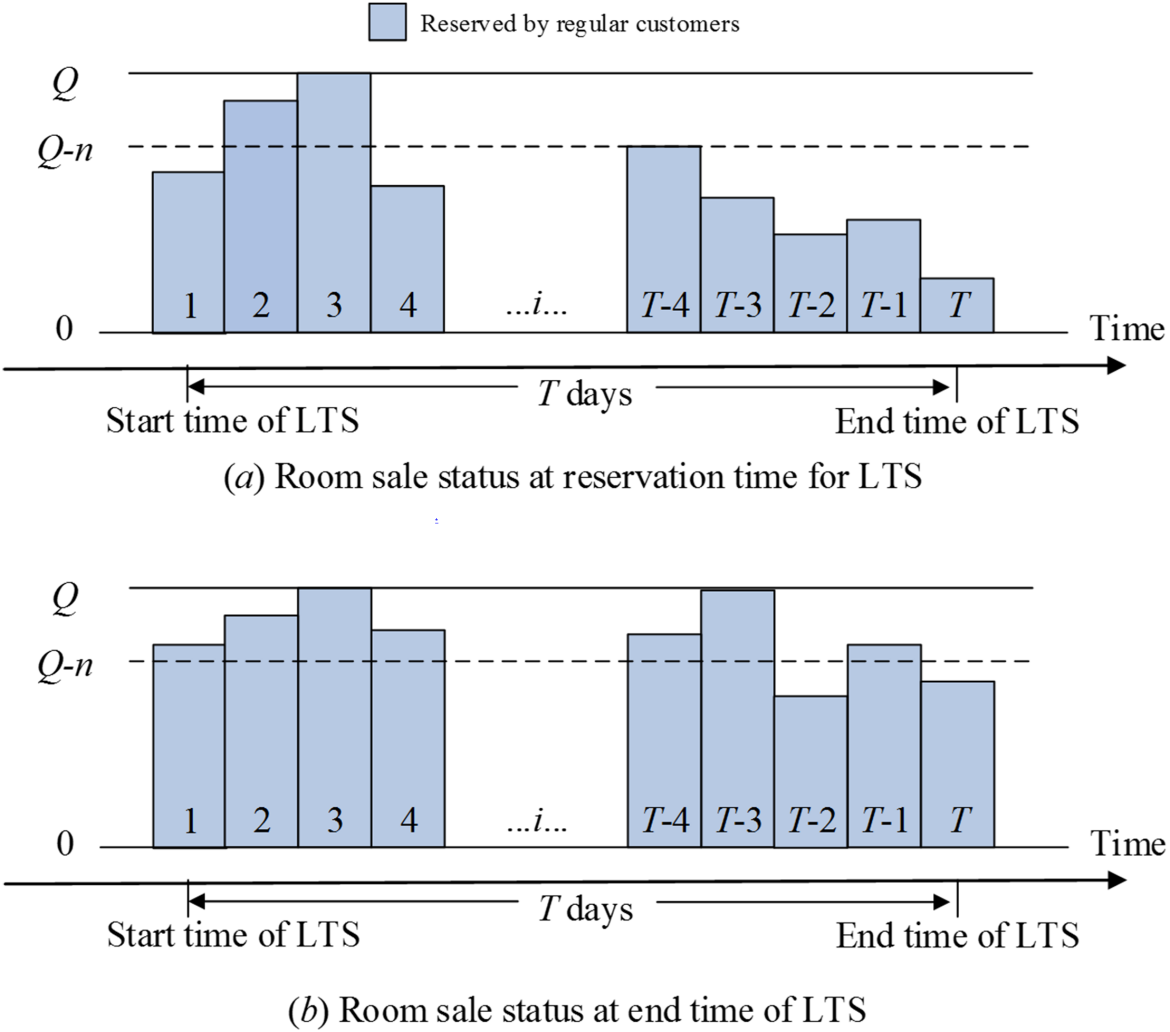
Room sale statuses without accepting LTS.

Generally, multi-item buying, such as LTS, may guarantee a specific amount of demand and generate opportunity income for a certain number of periods, meanwhile with risk of punishment incurred by overselling. To clearly describe the problem, we take hotels industry as an example, and consider a scenario under which the LTS customer intends to reserve *n* rooms for *T* day before *T*_0_ days from the start time of LTS. As Fig 1(*a*) shows, at the reservation time for LTS, all of the rooms on the third day have been sold; meanwhile, rooms are available on other days. The hotel should consider the room sale status at the reservation time for LTS and predict the room sale status after the ending date of LTS. The consideration of these times facilitates the comparison of expected profits during LTS according to two situations.

**Fig 2:**
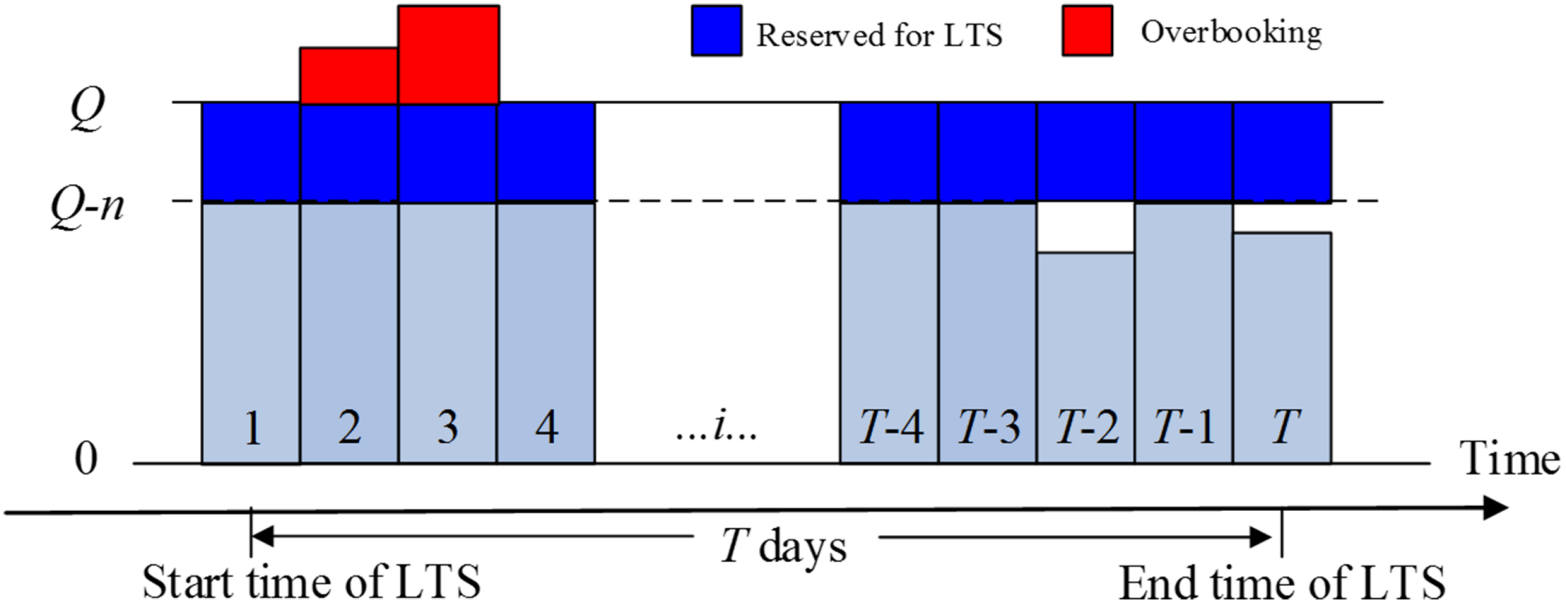
Room sale statuses with accepting LTS.

The room sale status at the ending date of LTS without accepting LTS is presented in Fig 1(*b*). Rooms on dates 3 and *T*-3 are sold out; by contrast, rooms are available on the other days. By comparing the two statuses in Figs 1(*b*) and 2, we find that these available rooms will incur opportunity losses, as Fig 2 shows. The marginal profit of each sold room is considerable, and the unit variable cost is lower than the high fixed cost [8]. Specifically, (1) for date *T*-3, the room sale statuses under two situations are the same by simply reserving *n* rooms for the LTS customer instead of regular customers. (2) For dates 1, 4, *T*-4, and *T*-1, the hotel will lose some regular customers, but LTS customers will replenish the empty rooms. (3) For date *T*-2 and *T*, the hotel will expand the room sales and not lose any regular customer.

Evidently, if the hotel reserves rooms for LTS customers, a number of rooms will be overbooked, such as dates 2 and 3 in Fig 2. The hotel must compensate the customers who have booked rooms on the two dates for the cancellations of reservations. In practice, many hotels will upgrade the room type free or pay more money than the room price to those customers with overbooking.

Considering opportunity losses, hotels with worse-than-expected sale status are motivated to accept LTS, though this decision will incur a penalty cost. However, by experiencing the reservation process for LTS with a popular online travel agency in China (e.g., www.ctrip.com) and the official website of a five-star hotel (e.g., www.wenyihotel.cn), we find that hotels will accept the request for LTS only if rooms are available for the entire LTS. By contrast, if no room is available even for one day within the LTS, the hotel will not reserve rooms for LTS customers due to the penalty cost incurred with overbooking [9–12]. Nevertheless, accepting the request for LTS can guarantee a specific demand and generate opportunity income, especially when the demand for rooms is lower. Consequently, the condition for hotels accepting the request from LTS customers is crucial.

In order to obtain maximal profit for a certain number of periods, service providers should make optimal decisions with trade-off between opportunity incomes obtained by multi-item buying and penalty cost incurred by overselling. For instance, hotels should decide whether to accept the request from LTS customers. However, few researchers focus on optimal decisions for hotels that simultaneous face LTS and overbooking.

To fill this gap in the literature, this study proposes a multi-period optimization model to describe the trade-off between opportunity incomes obtained with LTS and the penalty costs caused by overbooking. We aim to (1) determine the optimal decision rule for hotels facing LTS requests, and (2) analyze the effects of duration of stay, number of rooms for LTS, reservation time for LTS, and unit penalty cost with overbooking on the decision of the hotel.

To the best of our knowledge, this is the first research paper which attempts to explore the trade-off between the opportunity income incurred with LTS and penalty cost caused by overbooking. The rest of this paper is organized as follows. We provide literature review in the following section to describe the research gap and the necessity of the present research. Then the next section describes the problem, and gives the demand function and some assumptions. The optimal decisions for hotels with LTS and overbooking are explored in its following section. Numerical illustrations are provided in the last but one section, and the last section makes a conclusion for this paper and presents the problems of LTS to be solved in the future research.

## Literature Review

Three distinct streams of related literature should be considered, long-term stay, overbooking and multi-period optimization model in service industry.

### Long-term stay

Along with economic development and tourism popularity, LTS in a hotel is very common in reality. However, few researcher pay attention to this request from LTS customers. Generally, researchers set rooms on a particular day reserved by LTS customers as independent with other days. In fact, during LTS, rooms reserved by LTS customers should be regarded as a whole product.

To our best knowledge, at present, research on LTS principally focuses on pricing strategy. The paper by Ling et al. [2] is the first attempt to explore the optimal pricing strategy of hotel for long-term stay. They point out that a hotel with low occupancy rate provides LTS customers with low optimal room rate. However, they ignore the overbooking when the hotel accepts the request from LTS customers. With considering the overbooking, does the hotel accept LTS request? In this paper, we try to answer this question.

### Overbooking

In the past several decades, revenue management (RM) in tourism has received a great deal of attention both in practice and in academic research [13]. In order to obtain more profit, service providers adopt different strategies, such as overselling, dynamic pricing, and discount. There is a significant body of research in RM that proposes tactics, models, and methods to control these advance bookings. The oldest tactic is overbooking which improves the profit of service providers, such as American Airlines in 1990 [14] and US Airways in 2006 [15].

Beckmann [16] and Thompson [17] are the earlier researches about overbooking in RM. The paper by Shlifer and Vardi [18] is the earliest study about the overbooking decisions with considering the types of passengers. Following the previous studies about overbooking, many different models are formulated and analyzed, such as a multi-fare and single resource model with normal distribution [19], dynamic models with a single resource [20–26], overbooking model with multiple resources [27], and network revenue management problem with no-shows and/or cancellations [28–30].

Meanwhile, several researchers pay attention to the strategy of overbooking, and focus on optimal strategy in the context of overbooking in the airline industry [18, 23] and hotel industry [9, 12, 31–33], as well as the impact of overbooking on customers [34].

A common assumption in those models is that overbooking may incur penalty cost for service providers [9–12]. However, few reserachers study the optimal decisions for hotels that simultaneous face LTS and overbooking.

### Multi-period optimization model

In order to obtain maximal profit for a certain number of periods, service providers should make optimal decisions with trade-off between opportunity incomes obtained by multi-item buying and penalty cost incurred by overselling. For instance, hotels should decide whether to accept the request from LTS customers. At present, many researchers present multi-period optimization models [35–42], and provide many approaches to solve models [43, 44].

However, few reserachers focus on multi-period optimal decisions for service providers with considering multi-item buying and capacity constraint. In other words, previous dynamic pricing policies ingonre the multi-item buying, and the risk of overbooking incurred by accept multi-item buying. In hospitality, researchers set rooms on a particular day reserved by LTS customers as independent with other days. In fact, during LTS, rooms reserved by LTS customers should be regarded as a whole product. Furthermore, the previous models ignore the penalty cost incurred by overbooking when service providers accept multi-item request [11, 12]. For instance, when the hotels should accept LTS request facing LTS and overbooking simultaneously?

To fill this gap in the literature, this study proposes a multi-period optimization model to describe the trade-off between opportunity incomes obtained with LTS and the penalty costs caused by overbooking.

## Problem Description

A hotel has *Q* identical rooms and its daily variable cost per occupied room is *c*. The hotel customers are divided into two groups, namely, regular customers who reserve rooms only for one day and LTS customers who reserve rooms for a number of days. For convenience, we assume that (1) each regular customer reserves one room one day, and (2) there is only one LTS customer who reserves *n* rooms for *T* days. The reservation lead time is *T*_0_ days before check-in time.

The parameters involved in this paper are provided as follows.

Decision variable:

*θ*: 0–1 variable, represent denying LTS and accepting LTS, respectively

Input and output parameters:

*Q* capacity of the hotel
*c*: daily variable cost per occupied room
*n*: number of rooms required by an LTS customer
*T*: duration of LTS
*T*_0_: reservation time for LTS before the start time of LTS
*q*_*t*_: number of customers at time *t* before the target day
*p*: room price
*a*_*t*_: sale saturate market demand of hotel rooms
*ε*_*t*_: random variable related with time
${\bar{q}}_{t}$: expected number of rooms sold from reservation time for LTS to the ending date of LTS
*c*_*p*_: penalty cost per room per day caused by overbooking
Π_*nl*_: expected profit of hotel without accepting LTS during LTS
Π_*l*_: expected profit of hotel with accepting LTS during LTS
ΔΠ: expected incremental profit with accepting LTS during LTS

**Fig 3:**
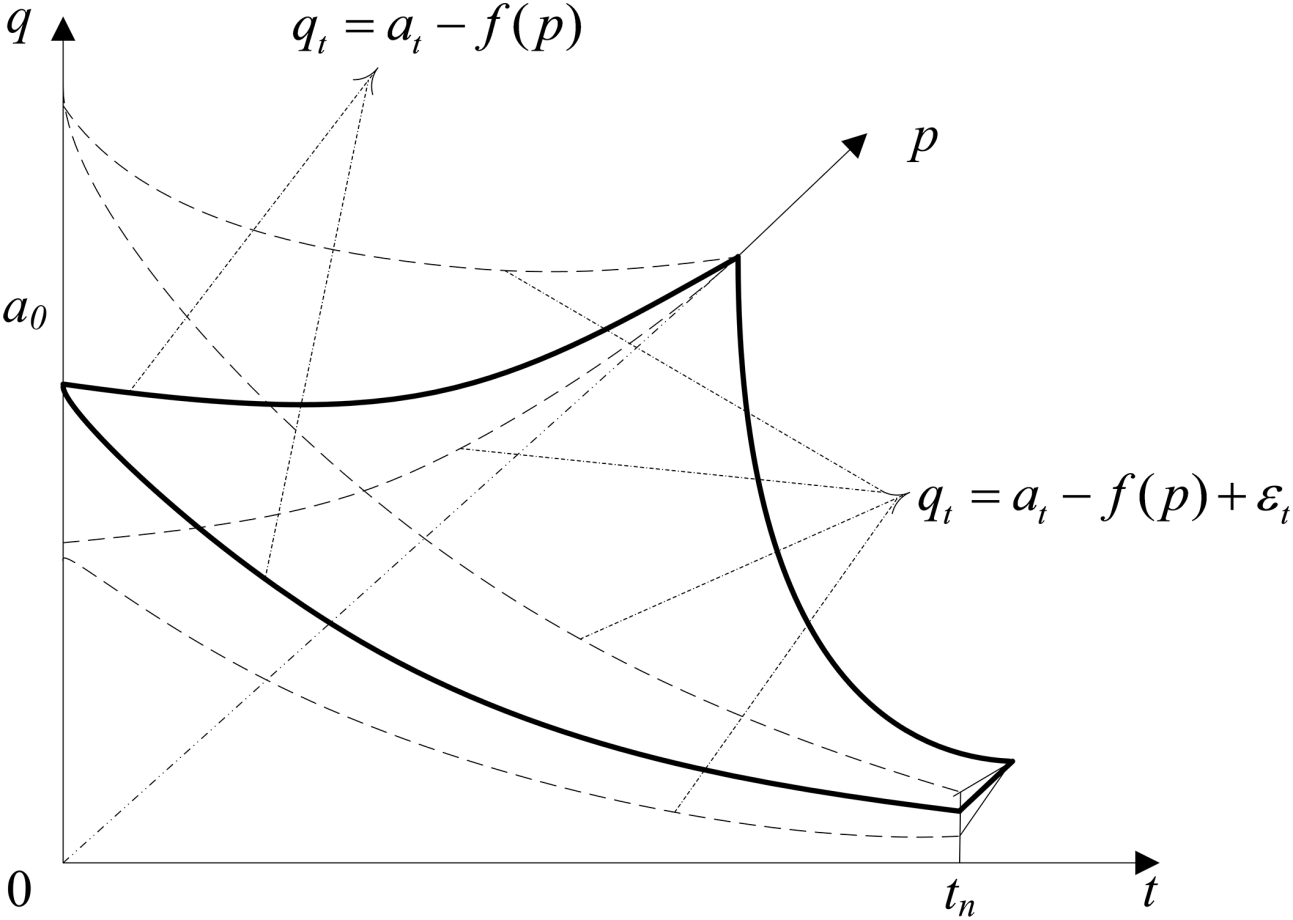
Example demand curves.

For hotel rooms on a particular day, the demand of customers is related to the room price and reservation time. We follow the demand model proposed by Guo et al. [38]. At time *t* before the target day, the demand curve shows the number of rooms demanded, *q*_*t*_, which is given by the decreasing relationship of the room rate *p*, that is, *q*_*t*_ = *a*_*t*_-*f*(*p*), where *a*_*t*_ = *g*(*a*_0_,*t*) is the sale saturate market demand of hotel rooms, increasing with the time closing to the target day; and *f*(*p*) is an increasing function of *p*, in relation to consumer sensitivity. However, due to the uncertainty, we regard each room at a particular day as a number of limited products, and the demand of customers of the hotel each day is an independent, identically distributed random variable. The room demand per day can be described as

$$q_{t}=a_{t}-f(p)+\varepsilon_{t},$$ (1)

where *ε*_*t*_ is a random variable related with time (see Fig 3).

**Fig 4:**
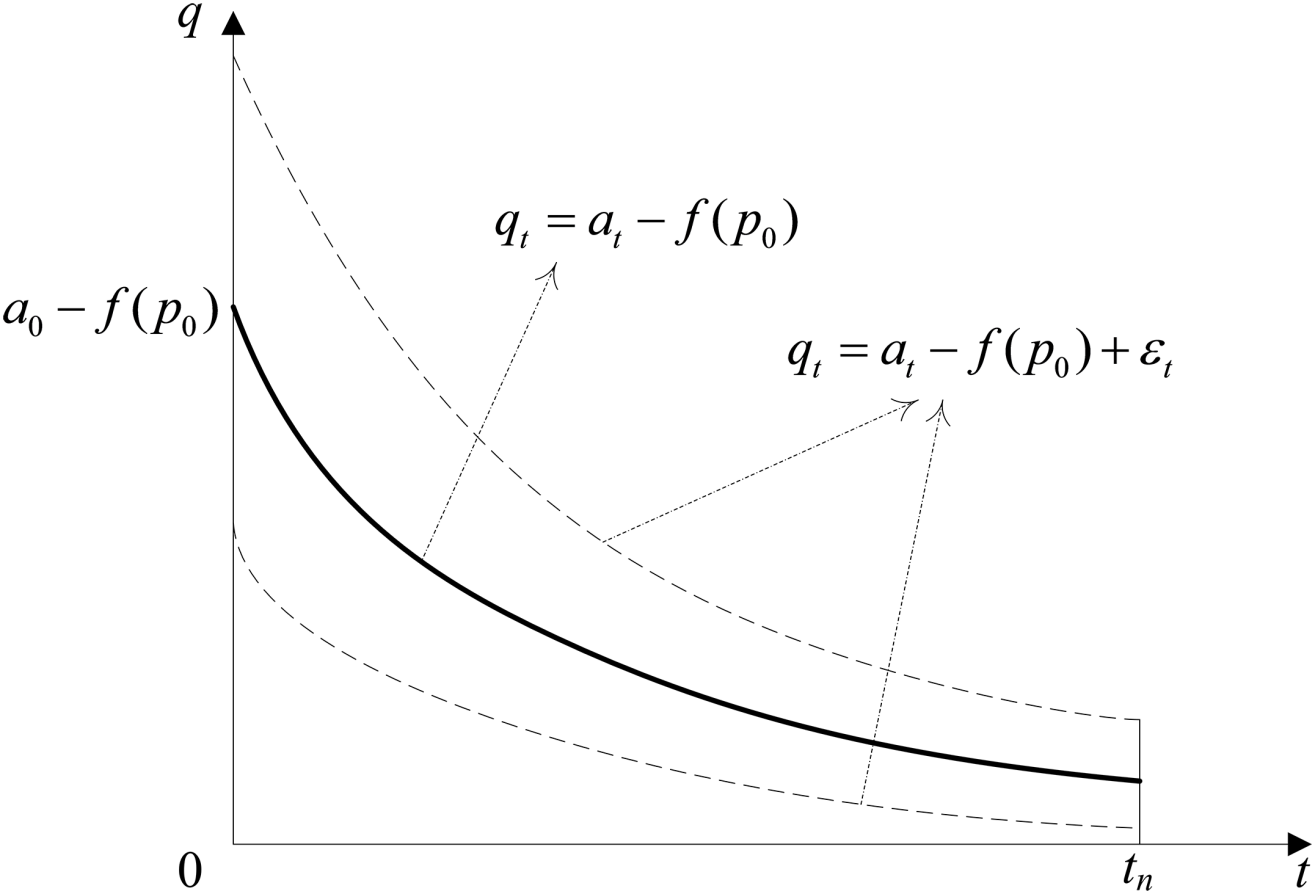
Projections of the demand curves in *t*0*q* coordinate system.

To clearly describe the problem, we obtain the projection of the demand curves in *t*0*q* coordinate system as shown in Fig 4. Fig 4 shows the demand curves if the price is constant, *p*_0_.

## Models and Analysis

In this paper, we discuss the optimal marketing decision for service providers with multi-period buying (LTS in hotels industry) and overbooking by taking hotels industry as an example. The LTS customer intends to reserve *n* rooms of the hotel for *T* days, and the reservation lead time is *T*_0_ days before check-in time. Accepting the request for LTS guarantees a specific demand and opportunity income, especially under the condition of lower demand, but it may incur penalty cost with overbooking. Then, the hotel should decide whether to accept the requirement from the LTS customer by comparing the expected profits during the LTS under two situations. To obtain expected profits, the hotel should consider the room sale status at the reservation time for LTS and predict the room sale status after the ending date of LTS.

### Modeling the expected profit without LTS

First, we consider the situation without accepting LTS. At the reservation time for LTS, the number of rooms that have been reserved at *i*th day of LTS is denoted as $q_{T_{0}+i-1}$, where *i* = {1,2,…,*T*}. For example, at the start date, the hotel has sold $q_{T_{0}}$ rooms, and sold $q_{T_{0}+2}$ rooms on the third day of LTS.

The hotel also needs to predict the room sale status after the ending date of LTS. We use ${\bar{q}}_{T_{0}+i-1}$ to indicate the number of rooms expected to be reserved at *i*th day of LTS. Then, without accepting LTS, the expected profit of the hotel during the LTS can be expressed as follows,

$$\Pi_{nl}=\sum_{i=1}^{T}\left(q_{T_{0}+i-1}+{\bar{q}}_{T_{0}+i-1})(p_{T_{0}+i-1}-c\right),$$ (2)

where $p_{T_{0}+i-1}$ is the room rate at *i*th day of LTS, and $q_{T_{0}+i-1}+{\bar{q}}_{T_{0}+i-1}\leq Q$.

### Modeling the expected profit with LTS

With accepting the request from the LTS customer, the hotel will reserve *n* rooms for the LTS customer. As previously discussed, this may incur penalty cost with overbooking, but bring considerable opportunity income. According to the room sale status at the reservation time for LTS and prediction of room sale status at the ending date of LTS, we can divide the room sale status at the ending date of LTS in four cases:

#### (1) $i\in I_{1}=\underset{i}{\text{arg}}\{Q-n<q_{T_{0}+i-1}\leq Q-{\bar{q}}_{T_{0}+i-1}\}$

In the first case, at the reservation time for LTS, the capacity of the hotel changes to *Q*-*n*. The dates at which the number of rooms that have been sold at the reservation time for LTS exceeds the new capacity (*Q*-*n*). This situation will induce overbooking as shown in dates 2 and 3 in Figs 1(*a*) and 2. The expected profit of the hotel during these days can be expressed as follows:

$$\begin{array}{l} \Pi_{l1}=\sum_{i\in I_{1}}^{}(p_{T_{0}+i-1}-c)Q-\sum_{i\in I_{1}}^{}(q_{T_{0}+i-1}-Q+n)c_{p}\\ \;=\sum_{i\in I_{1}}^{}\left[(p_{T_{0}+i-1}-c)Q-(q_{T_{0}+i-1}-Q+n)c_{p}\right] \end{array}$$ (3)

In formula (3), the former indicates the profit and the latter represents the penalty cost, where *c*_*p*_ is the penalty cost for each room per day. In practice, many hotels will upgrade the room type free or pay more money than the room price with overbooking.

#### (2) $i\in I_{2}=\underset{i}{\text{arg}}\{Q-n\geq q_{T_{0}+i-1}=Q-{\bar{q}}_{T_{0}+i-1}\}$

In the second case, the number of rooms that have been sold at the reservation time for LTS is less than the new capacity, and all of the rooms will be sold at the ending date of LTS, such as date *T*-3 in Figs 1(*b*) and 2. According to the prediction of room sale status after the ending date of LTS, we see that the hotel profits under two situations are the same by simply reserving *n* rooms for LTS customers instead of regular customers. Subsequently, the hotel can obtain the expected profit,

$$\Pi_{l2}=\sum_{i\in I_{2}}^{}(p_{T_{0}+i-1}-c)Q$$ (4)

#### (3) $i\in I_{3}=\underset{i}{\text{arg}}\{q_{T_{0}+i-1}\leq Q-n<q_{T_{0}+i-1}+{\bar{q}}_{T_{0}+i-1}<Q\}$

In the third case, the number of rooms that have been sold at the reservation time for LTS is less than the new capacity, and the number of rooms sold at the ending date of LTS is less than the old capacity, but larger than the new capacity, such as dates 1, 4, *T*-4, *T*-1 in Figs 1(*b*) and 2. According to the prediction of room sale status after the ending date of LTS, we see that by accepting LTS, all of the rooms will be sold. The hotel will lose some regular customers, but the LTS customer will replenish the empty rooms. Subsequently, the hotel can obtain the expected profit,

$$\Pi_{l3}=\sum_{i\in I_{3}}^{}(p_{T_{0}+i-1}-c)Q.$$ (5)

Although formulas (4) and (5) are the same, the opportunity incomes for hotels under two cases are different. In case (3), the opportunity income is 0, but it is positive in case (4).

#### (4) $i\in I_{4}=\underset{i}{\text{arg}}\{q_{T_{0}+i-1}+{\bar{q}}_{T_{0}+i-1}\leq Q-n\}$

In the fourth case, the number of rooms sold at the ending date of LTS is less than the new capacity, such as dates *T*-2 and *T* in Figs 1(*b*) and 2. Hence, the number of rooms sold at these days is relatively small. By accepting LTS, the hotel can obtain more profit. The hotel can obtain the expected profit,

$$\Pi_{l4}=\sum_{i\in I_{4}}^{}\left(p_{T_{0}+i-1}-c)(q_{T_{0}+i-1}+{\bar{q}}_{T_{0}+i-1}+n\right).$$ (6)

Based on the four cases, by accepting LTS, the expected profit of the hotel during the LTS can be expressed as follows,

$$\begin{array}{l} \Pi_{l}=\Pi_{l1}+\Pi_{l2}+\Pi_{l3}+\Pi_{l4}\\ \;=\sum_{i\in I_{1}}^{}\left[(p_{T_{0}+i-1}-c)Q-(q_{T_{0}+i-1}-Q+n)c_{p}\right]\\ \;+\sum_{i\in \{I_{2},I_{3}\}}^{}(p_{T_{0}+i-1}-c)Q+\sum_{i\in I_{4}}^{}\left(p_{T_{0}+i-1}-c)(q_{T_{0}+i-1}+{\bar{q}}_{T_{0}+i-1}+n\right) \end{array}$$

### Optimal decisions

The hotel should make optimal decisions by comparing the expected profits during the LTS under two situations. According to the discussions in 4.2, we find that a hotel faces penalty costs in case (1), and opportunity incomes in cases (3) and (4). Subsequently, the hotel will make the decision according to the profit-maximizing function of the system, which can be given as

$$\begin{array}{l} \begin{array}{cc} \text{max} & \Pi^{*}=(1-\theta )\Pi_{nl}+\theta \Pi_{l} \end{array}\\ \begin{array}{cc} s.t. & \;q_{T_{0}+i-1}+{\bar{q}}_{T_{0}+i-1}\leq Q \end{array}\\ \;\theta =\{0,1\} \end{array}.$$ (7)

Therefore, the optimal decision rules are as follows: (1) if Π_*nl*_≥Π_*l*_, the hotel should deny the request for LTS, and (2) if Π_*nl*_<Π_*l*_, the hotel should reserve *n* rooms for the LTS customer. Therefore, we only need to solve the formula ΔΠ = Π_*l*_-Π_*nl*_. Based on the expressions of the expected profits under two situations, the incremental profit incurred with LTS can be given as,

$$\begin{array}{l} \Delta \Pi \text{=}\Pi_{l}-\Pi_{nl}\\ \;=\sum_{i\in I_{1}}^{}\left[(p_{T_{0}+i-1}-c)Q-(q_{T_{0}+i-1}-Q+n)c_{p}\right]+\sum_{i\in \{I_{2},I_{3}\}}^{}(p_{T_{0}+i-1}-c)Q\\ \;+\sum_{i\in I_{4}}^{}\left(p_{T_{0}+i-1}-c)(q_{T_{0}+i-1}+{\bar{q}}_{T_{0}+i-1}+n\right)-\sum_{i=1}^{T}\left(q_{T_{0}+i-1}+{\bar{q}}_{T_{0}+i-1})(p_{T_{0}+i-1}-c\right) \end{array}$$ (8)

According to the analysis in 4.2, the profits in case (2) under two situations are the same, and formula (8) can be changed to

$$\begin{array}{l} \Delta \Pi \text{=}\Pi_{l}-\Pi_{nl}\\ \;=\sum_{i\in \{I_{1},I_{2},I_{3}\}}^{}\left(p_{T_{0}+i-1}-c)(Q-q_{T_{0}+i-1}-{\bar{q}}_{T_{0}+i-1}\right)+\sum_{i\in I_{4}}^{}(p_{T_{0}+i-1}-c)n\\ \;-\sum_{i\in I_{1}}^{}(q_{T_{0}+i-1}-Q+n)c_{p}\\ \;=\sum_{i\in \{I_{1},I_{2},I_{3}\}}^{}Q(p_{T_{0}+i-1}-c)-\sum_{i\in \{I_{1},I_{2},I_{3}\}}^{}\left(p_{T_{0}+i-1}-c)(q_{T_{0}+i-1}+{\bar{q}}_{T_{0}+i-1}\right)+\sum_{i\in I_{4}}^{}(p_{T_{0}+i-1}-c)n\\ \;-\sum_{i\in I_{1}}^{}(q_{T_{0}+i-1}-Q+n)c_{p}\; \end{array}$$ (9)

The first term is the total profit if all of the rooms are sold during the LTS, which can be easily determined by the hotel. The second term is the expected total profit without accepting LTS during the LTS, which is difficult to accurately ascertain for the hotel managers due to the uncertainty. The third term is the total penalty cost with overbooking, which can be easily identified by hotel managers at the reservation time for LTS. By solving model (9), we can obtain the optimal decision rules as follows,

$$\theta^{*}=\left\{ \begin{array}{l} 1,\;if\;c_{p}<\frac{\sum_{i\in \{I_{1},I_{2},I_{3}\}}^{}\left(p_{T_{0}+i-1}-c)(Q-q_{T_{0}+i-1}-{\bar{q}}_{T_{0}+i-1}\right)+\sum_{i\in I_{4}}^{}(p_{T_{0}+i-1}-c)n}{\sum_{i\in I_{1}}^{}\left(q_{T_{0}+i-1}-Q+n\right)}\\ 0,\;if\;c_{p}\geq \frac{\sum_{i\in \{I_{1},I_{2},I_{3}\}}^{}\left(p_{T_{0}+i-1}-c)(Q-q_{T_{0}+i-1}-{\bar{q}}_{T_{0}+i-1}\right)+\sum_{i\in I_{4}}^{}(p_{T_{0}+i-1}-c)n}{\sum_{i\in I_{1}}^{}\left(q_{T_{0}+i-1}-Q+n\right)} \end{array}\right.$$ (10)

***Proposition 1.** For hotels, the optimal marketing decisions with LTS and overbooking are as follows*,

1. *If the unit penalty cost caused by overbooking satisfies the condition*, *that is*,
  $$c_{p}<\left(\sum_{i\in \{I_{1},I_{2},I_{3}\}}\left(p_{T_{0}+i-1}-c)(Q-q_{T_{0}+i-1}-{\bar{q}}_{T_{0}+i-1}\right)+\sum_{i\in I_{4}}(p_{T_{0}+i-1}-c)n\right)/\sum_{i\in I_{1}}\left(q_{T_{0}+i-1}-Q+n\right),$$
  *hotels should reserve n rooms for LTS customers.*
2. *Otherwise*, *hotels should deny the requirement from LTS customers*.

Proposition 1 provides suggestions for hotels on the decisions when faced with the LTS requirements. The proposition clearly demonstrates that the decisions are related with unit penalty cost, number of rooms required by LTS customers, duration of LTS, and expected profit during LTS, and number of rooms overbooked at the reservation time for LTS.

(1) At the reservation time for LTS, the less number of rooms overbooked with LTS will increase the inclination of the hotel to accept LTS due to the smaller penalty cost caused by overbooking. (2) The more rooms the LTS customer requires, the less the number of rooms will be overbooked with LTS. The hotel is more inclined to accept LTS due to the smaller penalty cost caused by overbooking. (3) The smaller the expected profit during LTS is, the hotel becomes more inclined to accept LTS due to the opportunity income incurred by LTS. (4) The longer the reservation time for LTS before check-in time will reduce the number of rooms sold during LTS (as shown in Figs 3 and 4); thus, the hotel is more inclined to accept LTS.

## Numerical Experiments

**Fig 5:**
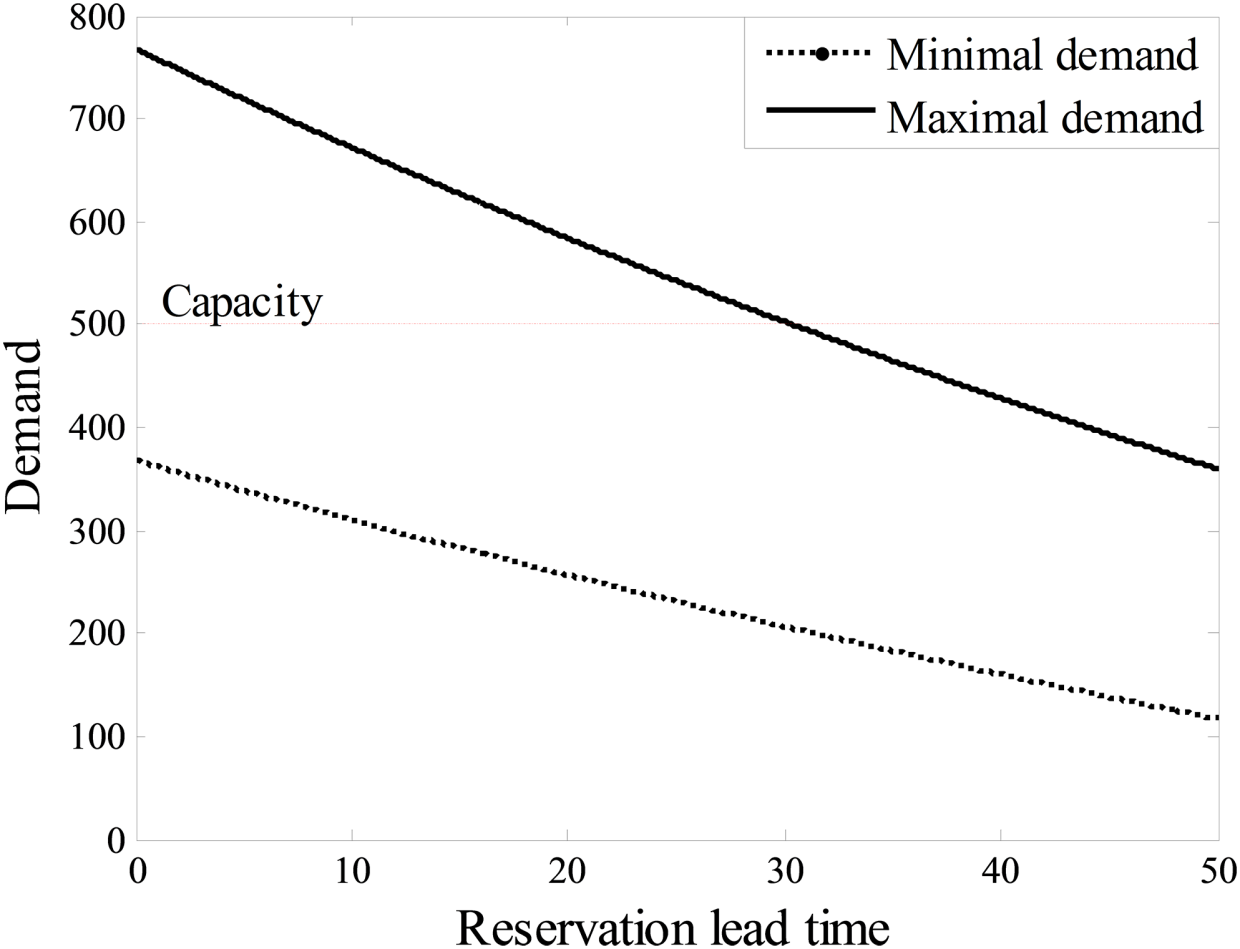
Hotel room demand based on lead time

To illustrate the optimal decision rule and explore the effects of the parameters on optimal decisions, we present the results of numerical experiments in this section. We assume that the hotel has *Q* = 500 rooms, and the variable cost of each room per day is *c* = 50. The fixed cost per day is ignored. In addition, for obtaining the room sale statuses under different situations, we adopt a simple algorithm to generate random numbers to represent the demand on each day by meeting the following demand function,

$$q_{t}=1000e^{-0.008t}-180\text{ln}p\pm 200e^{-0.01t},$$ (11)

which is similar to demand function proposed in Guo et al. [38]. We consider that demand on one day changes within a certain range, and set the room price as a constant value at 250. In reality, hotels charge the same price during a number of days. For instance, from January 22 to February 3, 2015, V Hotel Lavender in Singapore charges $104.2 per room, and the Upper House in Hong Kong charges $471.2 per room. The demand function can be changed to

$$q_{t}=1000e^{-0.008t}-180\text{ln}250\pm 200e^{-0.01t},$$ (12)

which is shown in Fig 5.

### Base example

In this section, we present a base numerical example to illustrate the decision rule. The LTS customers intend to reserve 10 rooms of the hotel for 10 days, and the reservation lead time is 10 days before check-in time (i.e., *n* = 5, *T* = 10, and *T*_0_ = 10). Based on demand function (12), we know the range of fluctuation of the demand on each day. We subsequently select the range from 10 to 20 in Fig 5. We use a simple random function to obtain the room sale status during LTS at the reservation time for LTS, and the expected room sale status at the ending date of LTS (see Table 1).

**Table 1:** Room sale status during LTS without accepting LTS.

Date of LTS	1	2	3	4	5	6	7	8	9	10
Max demand	672	663	654	645	636	627	618	609	601	592
Min demand	310	304	299	293	288	283	277	272	267	261
Demand at reservation time for LTS	500	487	450	408	343	354	500	449	338	302
Demand at ending date of LTS	500	491	480	500	470	489	500	490	479	488

From Table 1, we see that (1) on dates 1 and 7, all of the rooms are sold at the reservation time for LTS. (2) At the ending date of LTS, on date 4, the expected demand is 500, whereas rooms are available on other days. In addition, if the hotel accepts the request from LTS customer, penalty costs will be incurred for 20 rooms.

**Fig 6:**
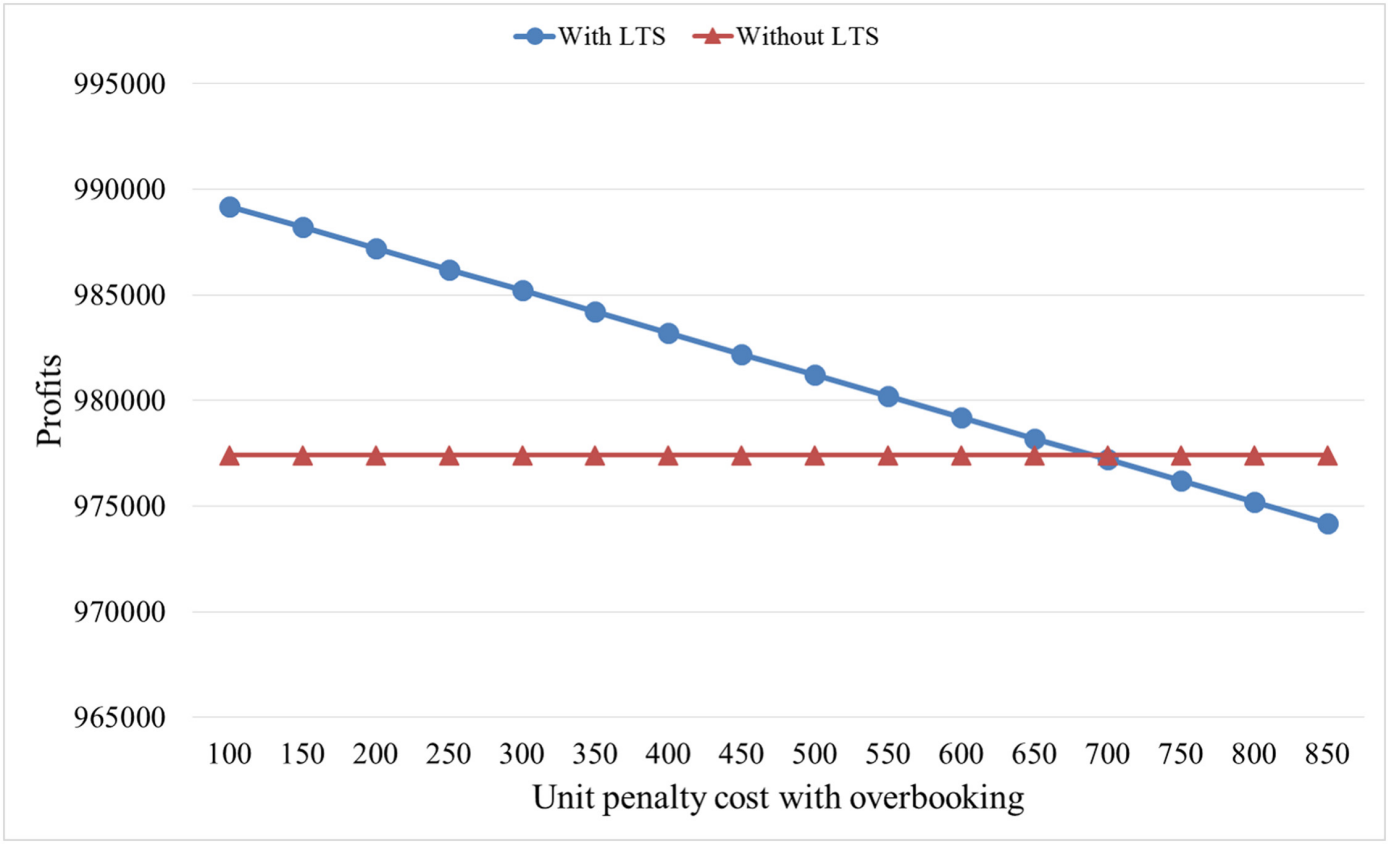
Hotel profits under different unit penalty costs.

According to proposition 1, we can obtain hotel profits under two situations with different unit penalty costs with overbooking, as shown in Fig 6.

From Fig 6, the hotel should accept LTS if the unit penalty cost is less than 690, and if the unit penalty cost is larger than 690, the hotel should deny LTS. Generally, the unit penalty cost is set as the twice room price (i.e., 500). By calculating if the penalty cost is 500, we obtain the expected profit without accepting that LTS is 977,400, and the expected profit with accepting LTS is 981,200.

In practice, the penalty cost incurred with overbooking, which is related with consumer type, such as old, new, and business customers, as well as tourists, takes several forms, such as paying money, loss of loyalty, and harming reputation. Hence, hotels should make decisions by considering the type of customers who reserve rooms earlier to reduce the loss of reputation.

### Effects of parameters about LTS on optimal decisions

In this section, we explore the effects on the optimal decisions of the number of rooms required by LTS, duration of LTS, and reservation time for LTS.

#### (1) Number of rooms required by LTS

**Fig 7:**
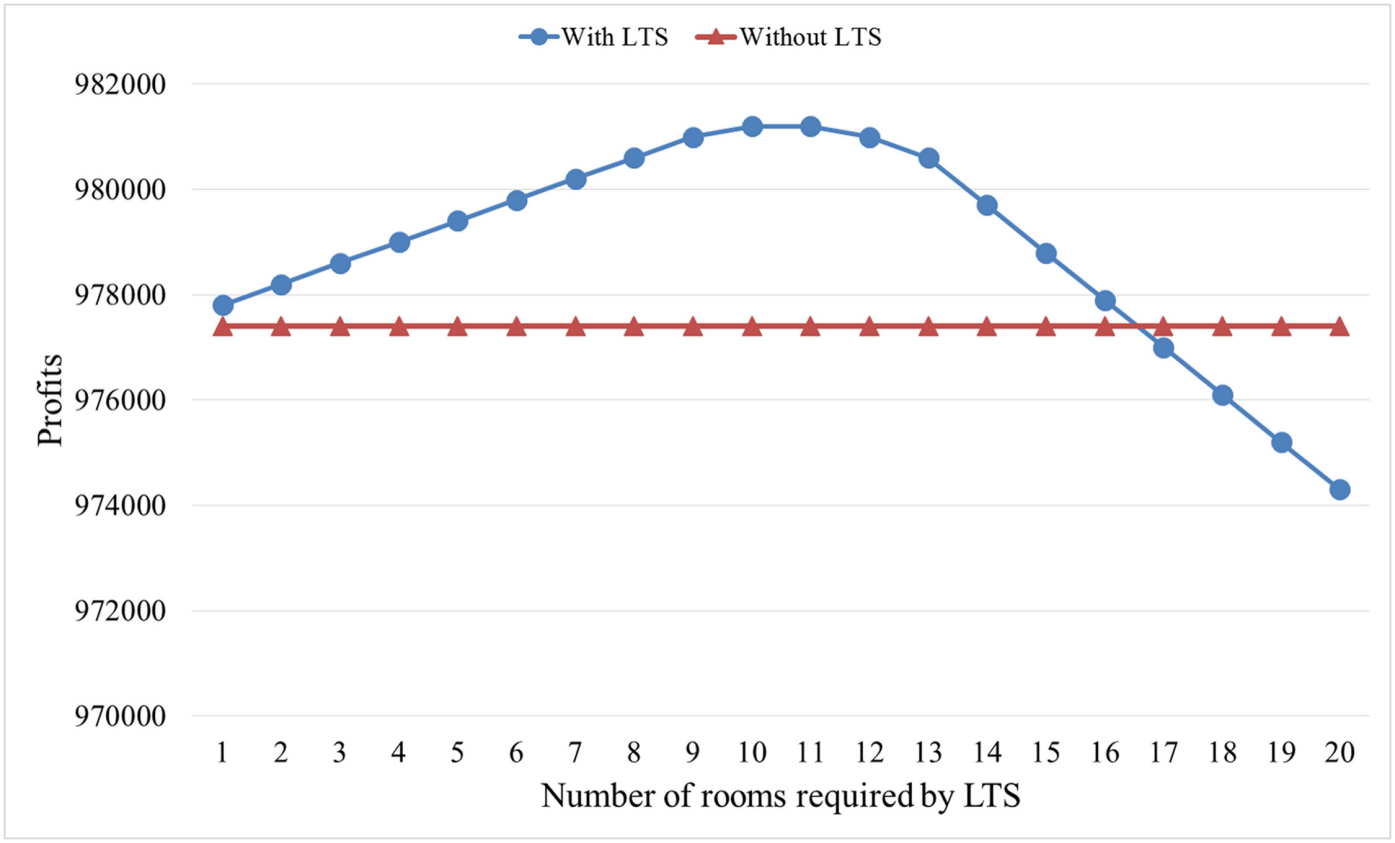
Effects of the number of rooms required by LTS.

In this example, the LTS customers intend to reserves *n* rooms of the hotel for 10 days, and the reservation lead time is 10 days before check-in time, that is, *n* = {1,2,…,20}, *T* = 10, and *T_0_* = 10. In addition, the unit penalty cost is set as twice that of room price (i.e., 500). We still use the room sale statuses under two situations in Table 1. By calculating, we obtain the expected profit without accepting LTS and the expected profit by accepting LTS under different numbers of rooms required by LTS, as shown in Fig 7.

From Fig 7, we can determine that (1) if the number of rooms required by LTS is less than 16, then the optimal decision of the hotel is to accept this request. However, if the number of rooms required by LTS is more than 16, then the hotel should deny the LTS customer by showing that the hotel has full reservations. (2) If the number of rooms required by LTS is less than 10, then the hotel profit with LTS increases in this number. However, hotel profit with LTS decreases if this number is larger than 10. In practice, hotels should make decisions by considering the number of rooms required by LTS to obtain more profit.

#### (2) Duration of LTS

The LTS customers intend to reserve 10 rooms of the hotel for *T* days, and the reservation lead time is 10 days before check-in time, that is, *n* = 10, *T* = {1,2,…,10}, and *T_0_* = 10. In addition, the unit penalty cost is set as twice that of the room price (i.e., 500). We still use the room sale statuses under two situations in Table 1. By calculating, we obtain the expected profit without accepting LTS and the expected profit by accepting LTS under different durations of LTS, as shown in Table 2. In this case, optimal decision 0 indicates denying LTS, whereas decision 1 signifies accepting LTS.

**Table 2:** Optimal decisions with different durations of LTS.

Duration of LTS	Profits with LTS	Profits without LTS	Optimal decision
1	95000	100000	0
2	195000	198200	0
3	293000	294200	0
4	393000	394200	0
5	489000	488200	1
6	588800	586000	1
7	683800	686000	0
8	783800	784000	0
9	883600	879800	1
10	983200	977400	1

From Table 2, if the durations of LTS are 1, 2, 3, 4, 7, and 8 days, then the hotel should deny LTS; however, if the durations of LTS are 5, 6, 9, and 10 days, then the optimal decision of the hotel is to reserve rooms for LTS customers.

According to the preceding analysis, on the first and seventh days, all of the rooms are sold at the reservation time for LTS. In other words, this decision will incur penalty costs for 20 rooms. Through a combination of this condition and optimal decisions in Table 3, we find that between two dates with overbooking and with the longer of duration of LTS, the hotel will change decisions from deny to acceptance, such as dates 5 and 6 in Table 2.

**Table 3:** Room sale statuses with different reservation times for LTS.

Date of LTS		1	2	3	4	5	6	7	8	9	10
Demand at reservation time for LTS	*T*_0_ = 0	500	491	475	499	435	450	500	470	420	405
	*T*_0_ = 1	500	491	475	498	422	442	500	465	405	389
	*T*_0_ = 2	500	491	474	497	411	431	500	460	381	374
	*T*_0_ = 3	500	488	470	496	402	423	500	455	369	367
	*T*_0_ = 4	500	488	462	495	394	411	500	452	360	352
	*T*_0_ = 5	500	488	458	484	382	396	500	451	352	337
	*T*_0_ = 6	500	488	455	463	371	382	500	450	347	328
	*T*_0_ = 7	500	487	453	441	363	378	500	450	344	321
	*T*_0_ = 8	500	487	452	425	355	367	500	449	341	314
	*T*_0_ = 9	500	487	452	415	349	359	500	449	339	308
	*T*_0_ = 10	500	487	450	408	343	354	500	449	338	302
Demand at ending date		500	491	480	500	470	489	500	490	479	488

#### (3) Reservation time for LTS

In this example, the LTS customers intend to reserve 10 rooms of the hotel for 10 days, and the reservation lead time is *T_0_* days before check-in time, that is, *n* = 10, *T* = 10, and *T_0_* = {0,1,2,…,10}. Room sale statuses at different reservation times for LTS vary. For instance, if *T_0_* is 2, then we should select the range from 2 to 12 in Fig 5, whereas we should select the range from 5 to 15 if *T_0_* is 5. We subsequently use a simple random function to obtain the room sale status during LTS at the reservation time for LTS, and the expected room sale status at the ending date of LTS (see Table 3).

**Fig 8:**
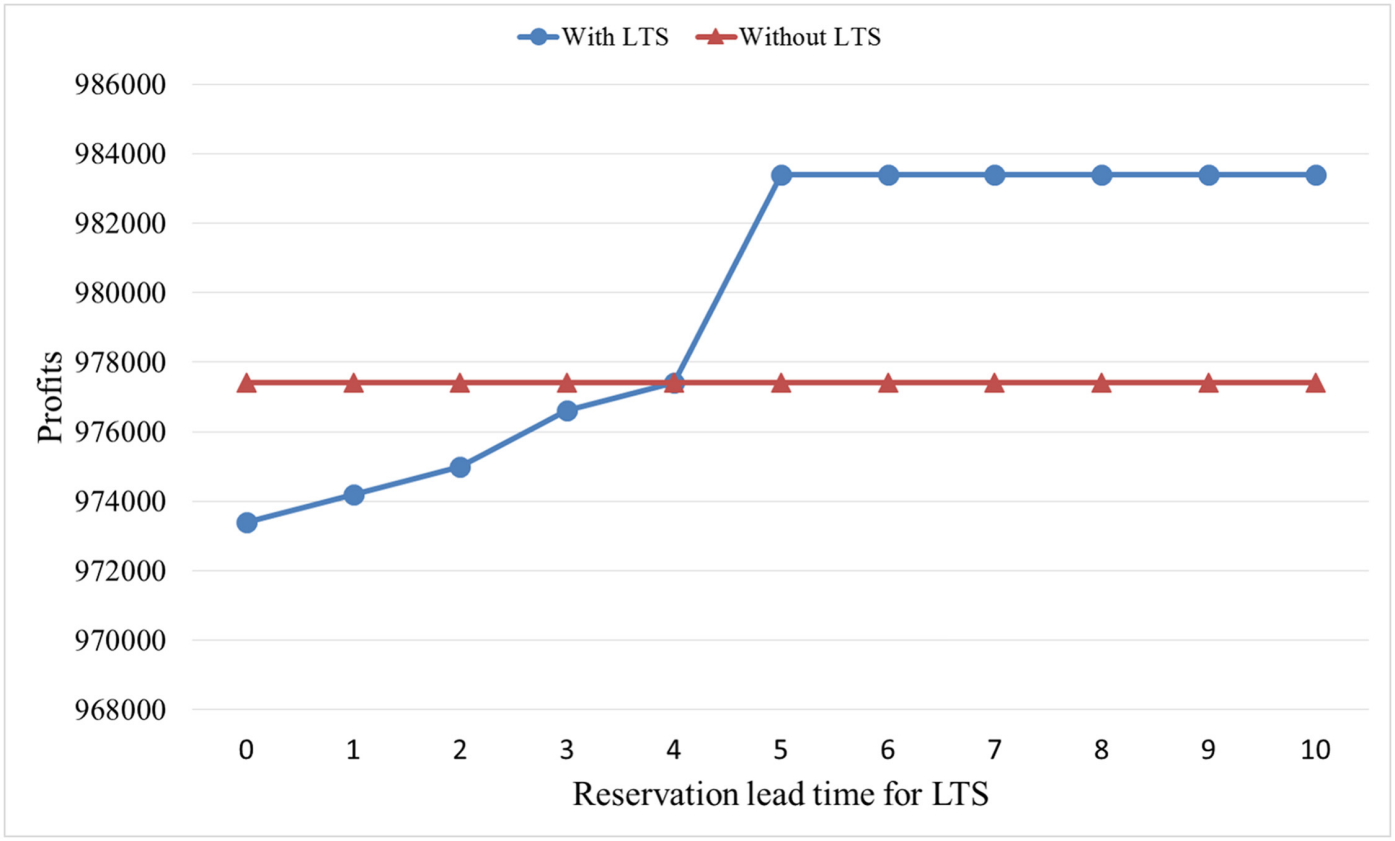
Effects of reservation time for LTS.

Table 3 indicates that a shorter reservation time will increase the number of rooms sold on each day. This situation will incur more penalty costs with overbooking. For example, if the LTS customer reserves rooms at two days before check-in time, then the number of rooms sold on the second and fourth days will exceed the new capacity, 490. Subsequently, penalty costs will be incurred for 28 rooms. We set the unit penalty cost at 600. By calculating, we determine the expected profit without accepting LTS and the expected profit with accepting LTS with different reservation times for LTS, as shown in Fig 8.

From Fig 8, (1) hotel profits with LTS do not decrease in reservation lead time for LTS. (2) If the reservation time for LTS is shorter than four days, then the hotel should deny LTS. (3) If the reservation time for LTS is longer than four days, then the hotel should reserve rooms for the LTS customer. According to the demand function shown in Figs 3 and 4, a longer reservation time for LTS before check-in time will reduce the number of rooms sold during LTS; thus, the hotel will be more inclined to accept LTS due to a smaller penalty cost. In practice, for LTS customers, the early reservation of rooms is the best choice for LTS.

## Conclusions and Further Research

This study explored the trade-off between opportunity income incurred with multi-item buying and penalty cost caused by overbooking for a certain number of periods. From a theoretical perspective, the study contributes to the revenue management literature by formulating the optimal decision rule for service providers facing multi-item buying and overbooking. At present, many multi-period optimization models are proposed by many researchers, such as Robinson and Lakhani [35], Wernerfelt [36], Guo et al. [38], Cui et al. [41] and Liu and Zhang [42]. However, multi-period buying and capacity constraint (overbooking) are not considered in previous models. Taking hotels industry as an example, researchers set rooms on a particular day reserved by LTS customers as independent with other days. In fact, during LTS, rooms reserved by LTS customers should be regarded as a whole product. In this paper, we consider the penalty cost incurred by overbooking when service providers accept multi-item request. We find that the optimal decisions are related with unit penalty cost, number of rooms required by LTS customers, duration of LTS, and expected profit during LTS, and number of rooms overbooked during the reservation time for LTS.

In terms of industry practice, this study provides valuable suggestions on marketing strategy for service providers. In practice, many online reservation systems ignored the multi-period buying, such as LTS in hotels industry. However, accepting the request for LTS can guarantee a specific demand and generate opportunity income, especially when the demand for rooms is lower. In this paper, we explore the effects of duration of stay, number of rooms for LTS, reservation time for LTS, and unit penalty cost with overbooking on the decisions of hotels by considering LTS and overbooking. The findings indicate that (1) the expected profit of the hotel upon acceptance of LTS decreases in unit penalty cost. (2) The function between hotel profit with LTS and number of rooms required by LTS is represented by a downward parabola. (3) Between two dates with overbooking, with a longer duration of LTS, the hotel will change decisions from deny to acceptance. (4) With a shorter reservation time, the numbers of rooms sold increases each day, which could incur more penalty costs due to overbooking. (5) Longer reservation time for LTS before check-in time results in fewer rooms sold during LTS. Hence, the hotel is more inclined to accept LTS due to a smaller penalty cost. In practice, service providers should make decisions based on the types of customers, number of products required, and duration of multi-period to reduce the loss of reputation and obtain more profit. In addition, ordering earlier and improvement of power are better options for multi-period buying customers.

Finally, this study is a preliminary research that involves optimal decisions for hotels with LTS and overbooking. Several limitations, which could be streamlined into interesting directions for further research, are identified. First, we ignore the fluctuation of room price and discount for LTS. Second, we disregard the cancellation, which increases uncertainty. Although more challenges will be encountered in adhering to these directions, such initiatives would yield potentially interesting insights for the hotel industry.
